# Prediction of Geosmin at Different Depths of Lake Using Machine Learning Techniques

**DOI:** 10.3390/ijerph181910303

**Published:** 2021-09-30

**Authors:** Yong-Su Kwon, In-Hwan Cho, Ha-Kyung Kim, Jeong-Hwan Byun, Mi-Jung Bae, Baik-Ho Kim

**Affiliations:** 1 EcoBank Team, Division of Ecological Information, National Institute of Ecology, Seocheon, Busan 33657, Chungcheongnam-do, Korea; kwonys@nie.re.kr; 2 Department of Environmental Science, Hanyang University, Seoul 04763, Korea; crice5237@naver.com (I.-H.C.); hosang9022@naver.com (H.-K.K.); 3 Han-River Environment Research Center, National Institute of Environmental Research, Yangpyeong-gun, Incheon 12585, Gyeonggi-do, Korea; jh0130@korea.kr; 4 Department of Life Science, Hanyang University, Seoul 04763, Korea; 5 Biodiversity Research Team, Freshwater Biodiversity Research Bureau, Nakdonggang National Institute of Biological Resources, Sangju 37242, Gyeongsangbuk-do, Korea; mjbae@nnibr.re.kr

**Keywords:** taste-and-odor compound, off-flavor material, species distribution models, random forest, vertical difference

## Abstract

Geosmin is a major concern in the management of water sources worldwide. Thus, we predicted concentration categories of geosmin at three different depths of lakes (i.e., surface, middle, and bottom), and analyzed relationships between geosmin concentration and factors such as phytoplankton abundance and environmental variables. Data were collected monthly from three major lakes (Uiam, Cheongpyeong, and Paldang lakes) in Korea from May 2014 to December 2015. Before predicting geosmin concentration, we categorized it into four groups based on the boxplot method, and multivariate adaptive regression splines, classification and regression trees, and random forest (RF) were applied to identify the most appropriate modelling to predict geosmin concentration. Overall, using environmental variables was more accurate than using phytoplankton abundance to predict the four categories of geosmin concentration based on AUC and accuracy in all three models as well as each layer. The RF model had the highest predictive power among the three SDMs. When predicting geosmin in the three water layers, the relative importance of environmental variables and phytoplankton abundance in the sensitivity analysis was different for each layer. Water temperature and abundance of Cyanophyceae were the most important factors for predicting geosmin concentration categories in the surface layer, whereas total abundance of phytoplankton exhibited relatively higher importance in the bottom layer.

## 1. Introduction

Globally, cyanobacterial blooms as a result of abnormal growth of algae signify problems such as nutrient over-enrichment, modified hydrology, and poor management of water bodies [1]. These cyanobacterial blooms cause changes in various biological habitats of water bodies through deterioration of water quality. In addition, algal and cyanobacterial blooms degrade water quality in drinking water supply reservoirs by producing toxic and unpleasant taste-and-odor causing secondary metabolites, which ultimately cause public health concerns and lead to increased treatment costs for water utility companies [2]. In fact, Dodds et al. [3] reported that, in the U.S., US$813 million is spent annually on bottled water because of taste and odor problems, potentially linked to eutrophication, in the tap water supply. Furthermore, the potential annual value of losses in waterfront real estate, recreational water usage, and spending on recovery of threatened and endangered species due to eutrophication have been estimated to be US$2.2 billion annually for US freshwaters.

Most of the taste and odor events in drinking water caused by algal blooms are predominantly associated with microbial metabolites such as geosmin and 2-methylisoborneol (2-MIB) [4,5]. Although these compounds are not hazardous to human health, they render water aesthetically unpleasant and may result in subsided consumer trust [6,7,8]. Moreover, these compounds are generally stable and resistant to traditional water treatment processes like coagulation and sedimentation. Geosmin, which was first discovered in materials isolated from actinomycetes [9], is difficult to remove completely with conventional water treatment operations. However, unpleasant taste and odor caused by geosmin can be detected at even a few ng/L, therefore, a separate process that can treat trace amounts is required for its removal, such as adsorption onto powdered or granular activated carbon (GAC) [6,10,11].

Geosmin is the major taste-and-odor compound found in rivers and reservoirs during and after cyanobacterial blooms in Korea [5]. In fact, in 2011 and 2012, large amounts of Anabaena were found in the North-Han River watershed located upstream of an important water source, the Paldang Lake; a high concentration of geosmin, more than 1000 ng/L, was observed, which caused serious discomfort to the populace of the metropolitan areas supplied with the affected water [12,13]. As a result, the geosmin level was designated as a drinking water quality item required to be monitored in Korea and has since been managed below 20 ng/L [14]. The increase of geosmin in water sources is a problem not only in Korea, but also worldwide. Ma et al. [15] reported problems in drinking water supply due to toxins and taste-and-odor compounds caused by the outbreak of microcystis in 2007 at Lake Taihu, China. In addition, high concentrations of geosmin have been reported in natural water sources at concentrations of 400 ng/L in Japan [16], 86 ng/L in Spain [17], 4000 ng/L in Australia [18] and 3170 ng/L in South Africa [19].

In this sense, in order to manage geosmin in the water bodies worldwide, there has been a lot of research done to predict the occurrence or metabolite of geosmin based on various modelling techniques. For instance, Parinet et al. [20] and Sugiura et al. [21] compared multiple linear regressions (MLRs) and artificial neural networks (ANNs) for metabolite production modeling. In addition, multiple studies [2,4,7,22] on metabolites such as geosmin have used regression-based methods to relate geosmin concentrations to abiotic factors and/or diverse phytoplankton species. Meanwhile, two-dimensional (2D) hydrodynamic and water quality models [5] and three-dimensional (3D) hydrodynamic ecological models [23] have been used to predict the occurrence of algal blooms and algal-derived geosmin in Korea. However, most models developed in previous studies have been empirical and applicable only to specific water bodies. In addition, most studies have analyzed only particular species (e.g., cyanobacteria) in the surface layer of water bodies, even though water body characteristics can differ significantly with depth.

Therefore, in this study, we predicted the concentration of geosmin based on various factors including phytoplankton abundance, physicochemical factors and water quality factors at three different depths of lakes using three different machine-learning techniques. Our goals were as follows: (1) to select the most suitable model for predicting geosmin concentration in lakes; (2) to compare the occurrence patterns of geosmin at three different water depths; and (3) to identify the most important variables, in terms of environmental factors and phytoplankton abundance, influencing the occurrence of geosmin in lakes.

## 2. Materials and Methods

### 2.1. Ecological Data

**Figure 1:**
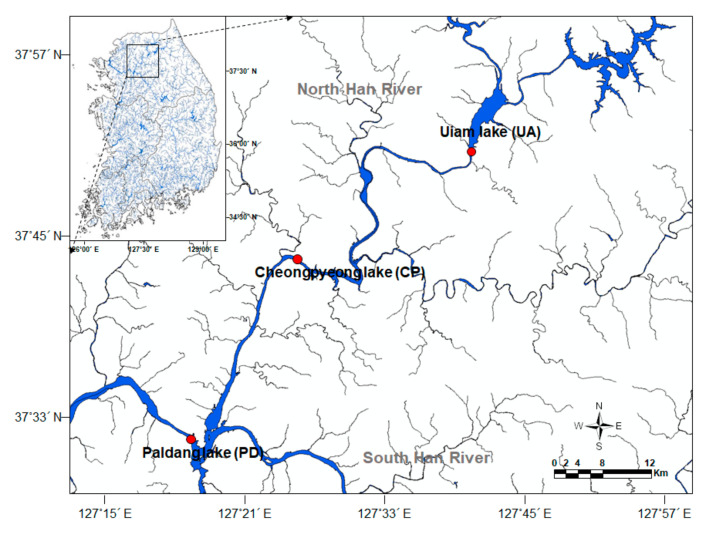
Locations of the study sites (●) in the Han River watershed of South Korea.

The mean annual temperature and the total amount of annual precipitation in the Han River watershed, which includes Uiam lake, Cheongpyeon lake and Paldang lake during the last 10 years was 10.5 °C and 1319 mm, respectively. Almost 70% of the total annual precipitation occurs from June to September (Korea Meteorological Administration, http://www.kma.go.kr/ (accessed on 17 September 2021)). The morphometric and hydrological characteristics of the three lakes are indicated in Table 1. The data for the phytoplankton community and the environmental variables were obtained from the Basic Environmental Research Program (Investigation of causes of off-flavor material production by harmful algae and management strategy) operated by the Han River Watershed Management Committee (HRWMC) and the Ministry of Environment (MOE), Korea. Samples were surveyed monthly at three major lakes (Uiam, Cheongpyeong, and Paldang lakes) located in the North-Han River watershed area from May 2014 to December 2015 (Figure 1). We conducted samplings 400 m upstream of the dam and samples were collected from three different layers (i.e., surface, middle, and bottom layers). Because of different water depth in the three lakes, the sampling interval among the three layers was different (Uiam lake: 8 m, Cheongpyeong lake: 13 m, and Paldang lake: 10 m). Some data could not be measured due to freezing of lakes (Uiam lake: January to February 2015; Cheongpyeong lake: November 2014 to February 2015, and December 2015; Paldang lake: December 2014 to February 2015) or missing surveys (April 2015).

Phytoplankton and environmental variables were surveyed according to the sampling protocol of HRWMC [12]. Water samples at each layer for the identification of phytoplankton species and measurement of cell densities were taken using a Van Dorn sampler (Halltech Environmental Inc., Guelph, ON, Canada) and stored in Whirl-Pak bags (250 mL), then fixed with Lugol’s solution (2% final concentration). Cell density was measured using a microscope (Axiostar plus; Zeiss, Jena, Germany) with a Sedgwick-Rafter counting chamber at 200–400× magnification. Phytoplankton were identified to the species level [24,25,26].

Water temperature, pH, DO, conductivity, and turbidity were measured in situ using a water quality logger (YSI-6600D, YSI Inc., Yellow Springs, OH, USA). Other variables, such as BOD, SS, TOC, TN, TP, and chlorophyll-*a*, were analyzed in the laboratory using standard methods [27] (Table 2). Geosmin was determined using Head Space-Solid Phase MicroExtraction (HS-SPME) and gas chromatography/mass spectrometry (GC/MS; 450-GC, 320-MS, Bruker, Billerica, MA, USA) [14]. A Polydimethylsiloxane (PDMS) fiber (47525-U, Supelco, Sigma-Aldrich, St. Louis, MO, USA) was used for the SPME, helium was the carrier gas, and a VF-5MS column 30 m in length and 0.25 mm in diameter was used for separation. Geosmin concentrations <1 ng/L were considered not detectable (ND).

### 2.2. Data Analysis

**Figure 2:**
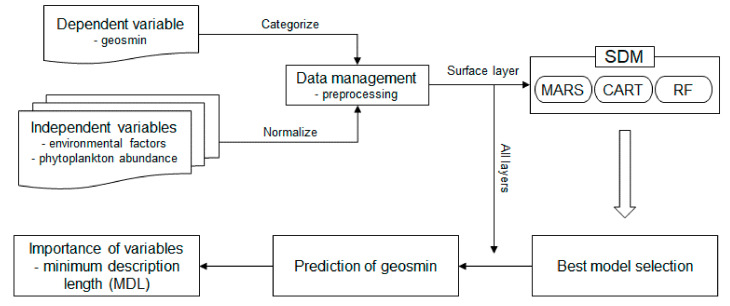
Flow chart of the modeling procedures to predict geosmin concentration categories based on phytoplankton abundance and environmental variables.

Geosmin concentration was categorized into four groups based on the boxplot method (A: <25%, B: 25–50%, C: 50–75%, D: >75%) (Table 3). To predict geosmin concentration categories based on phytoplankton abundance and environmental variables, we applied three representative machine learning techniques such as multivariate adaptive regression splines (MASR), classification and regression trees (CART), and random forest (RF) (Figure 2). These three different models were chosen by considering model complexity and error [28]. All the machine learning techniques were trained and tested based on 10-fold nested cross-validation (training:test = 9:1). [28]. A total of 47 samples from three lakes were used to construct the model.

After the learning process, each model’s performance was tested based on accuracy and area under an ROC curve (AUC) which was widely applied in ecology e.g., [29]. The accuracy computed the correct prediction rate between predicted and observed data [30]. AUC measures a model’s overall performance [30], and ranges from 0 to 1. As a rule of thumb, AUC values above 0.90 indicate excellent, values between 0.80 and 0.90 indicate good, values between 0.70 and 0.80 indicate fair, and 0.60–0.70 and the values below 0.60 indicate fail according to this model [31]. In each prediction model, the relative importance of independent variables for predicting geosmin concentration was evaluated using minimum description length (MDL), which measures the ability of an attribute to compress data [32]. The MDL values were rescaled to range from 0 to 100 to compare the relative importance of each environmental factor. Importance values provided by the algorithm were averaged after 10 repetitions. The prediction models were run in the R computing environment (https://cran.r-project.org/ (accessed on 8 July 2021)) with packages earth [33], rpart [34], and CORElearn [35] for the MARS, CART, and RF models, respectively.

To remove the effect of unit differences [36], all of the independent variables were normalized to the standard deviation of each variable using Formula (1) after natural log (ln (x + 1)) transformation.

(x − avg)/stdev,(1)

where x is a response variable, avg is the average of a response variable, and stdev is the standard deviation of a response variable. Before analyzing the data, all the outlier and extreme values by sampling error were deleted using the boxplot method [37].

## 3. Results

### 3.1. Relations between Geosmin and Phytoplankton Abundance and Environmental Variables

**Figure 3:**
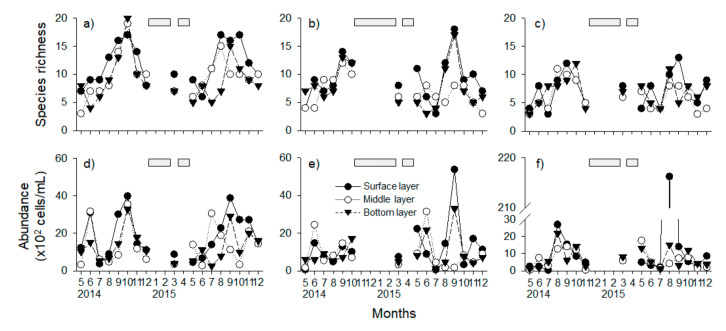
Temporal dynamics of the species richness and abundance of phytoplankton communities in three lakes—Uiam (**a**,**d**), Cheongpyeong (**b**,**e**), and Paldang (**c**,**f**)—from May 2014 to December 2015. Gray bars indicate data loss/missing data due to freezing conditions or missing survey.

Species richness and abundance of phytoplankton communities showed clear seasonal dynamics at the three different depths of each lake (except in winter, when data could not be collected due to freezing conditions) (Figure 3). Species richness and abundance of phytoplankton communities were highest at all depths between August and October, and especially in the surface layer during most of the sampling period. Meanwhile, the abundance of phytoplankton communities was the highest in the surface layer of Paldang lake in August 2015 (21,624 cells/mL). The changes of species richness and abundance of phytoplankton communities in Uiam Lake was a significant positive correlation among three layers except for the species richness in the middle and bottom layer (*r* > 0.50, *p* < 0.05), whereas Cheongpyeong Lake significantly correlates with species richness only between the surface and bottom layers (*r* = 0.70, *p* < 0.05).

**Figure 4:**
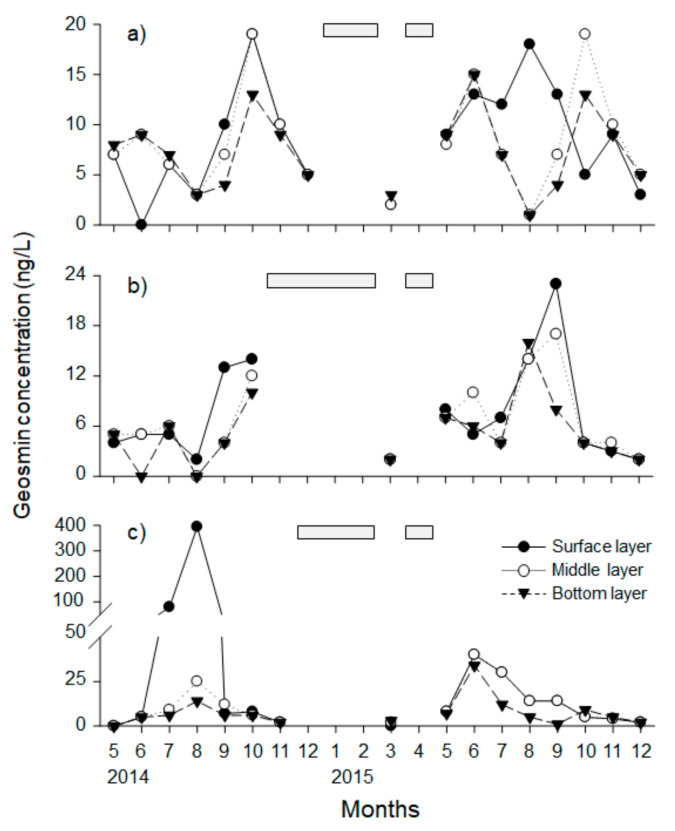
Temporal variation of geosmin at three different water depths in three lakes—(**a**) Uiam, (**b**) Cheongpyeong, (**c**) Paldang lake—from May 2014 to December 2015. Gray bar indicates data loss/missing due to freezing conditions or missing survey.

Geosmin concentration showed a similar pattern to that of phytoplankton communities, and was higher in summer (June to October) than other periods (Figure 4). In particular, the concentration of geosmin in Paldang Lake exhibited very high values of 394 ng/L and 80 ng/L, respectively, in August and July 2014, but all the rest showed a concentration below 40 ng/L. Meanwhile, the monthly average geosmin concentration was highest in the upper layer (18 ng/L) among three different depths, and the concentration decreased toward the bottom layer (7 ng/L). The changes of geosmin concentrations showed a significant positive correlation among three layers in Cheongpyeong Lake and Paldang Lake (*r* > 0.64, *p* < 0.05), and positively correlated between middle and bottom layer in Uiam Lake (*r* = 0.92, *p* < 0.05).

**Figure 5:**
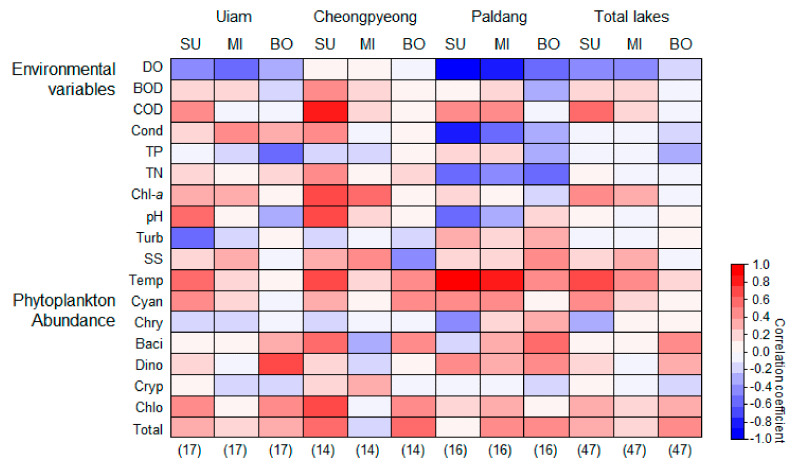
Correlation between geosmin and phytoplankton abundance and environmental variables. The color gradient (from −1 to 1) indicates Spearman’s correlation coefficients. The darker red indicates a higher positive correlation (*p* < 0.05), and darker blue indicates a higher negative correlation (*p* < 0.05). The numbers in parenthesis indicate the number of sampling sizes. Abbreviation of each variable indicated in Table 2. SU: surface layer, MI: middle layer, BO: bottom layer, and Total lake: pooled sample in each layer from three lakes.

We used Spearman’s rank correlation to evaluate the relationship of geosmin concentration with phytoplankton abundance and environmental variables (Figure 5). Geosmin concentration in the surface layer of the total lake (sum of the three lakes) was positively correlated with the abundance of Cyanophyceae (*r* = 0.44, *p* < 0.05) and Chlorophyceae (*r* = 0.32, *p* < 0.05), while the abundance of Bacillariophyceae (*r* = 0.39, *p* < 0.05), Dinophyceae (*r* = 0.34, *p* < 0.05), Chlorophyceae (*r* = 0.30, *p* < 0.05), and total phytoplankton (*r* = 0.42, *p* < 0.05) showed a positive correlation with geosmin concentration in the bottom layers of total lake (sum of the three lakes). Meanwhile, geosmin concentration was negatively correlated with the DO of the surface (*r* = −0.79, *p* < 0.05) and middle (*r* = −0.76, *p* < 0.05) layers in Paldang, and positively correlated with the water temperature in the surface layer of all lakes (Uiam lake *r* = 0.54, Cheongpyeong lake *r* = 0.59, Paldang lake *r* = 0.82, *p* < 0.05).

### 3.2. Prediction of Geosmin Concentration

To predict the geosmin concentration categories, we used three different SDMs—MARS, CART, and RF—according to environmental variables and phytoplankton abundance (Table 4). The RF model performed best in terms of both environmental variables and phytoplankton abundance (AUC > 0.910, accuracy > 0.680); in particular, the prediction of the category with the highest geosmin concentration with environmental variables showed the highest predictive power (AUC: 0.969, accuracy: 0.872). The prediction of the category with the highest geosmin concentration was more predictable in all three SDMs than the prediction of four categories. Meanwhile, MARS exhibited the worst predictive performance in the prediction of four categories (AUC: 0.623, accuracy: 0.447) and the prediction of highest geosmin concentration (AUC: 0.780, accuracy: 0.745) when only phytoplankton abundance data were used.

The RF model, which had the highest predictive power with environmental variables and phytoplankton abundance data among the three SDMs, was applied to the three different water layers (Table 5). All three different layers were well predicted by RF models using environmental variables and phytoplankton abundance (prediction accuracy > 0.610).

**Figure 6:**
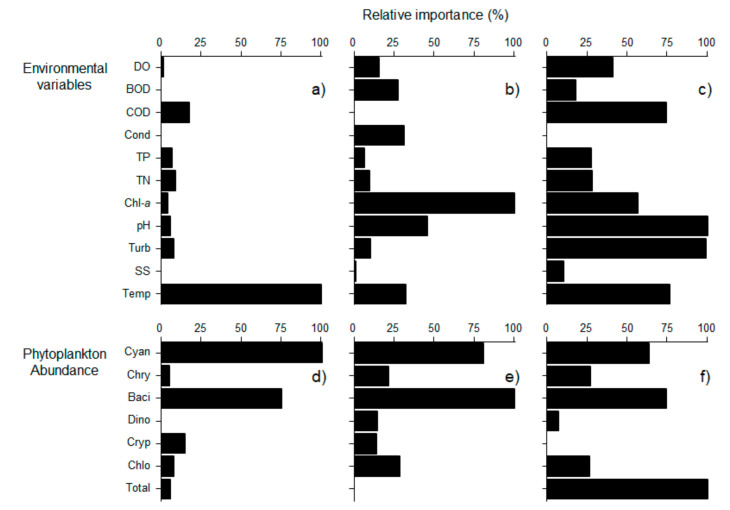
Relative importance (%) of environmental variables (**a**–**c**) and phytoplankton abundance (**d**–**f**) in predicting the four categories (A–D) of geosmin concentration in three different depths. Surface layer (**a**,**d**), Middle layer (**b**,**e**), and Bottom layer (**c**,**f**). Abbreviation of each variable indicated in Table 2.

**Figure 7:**
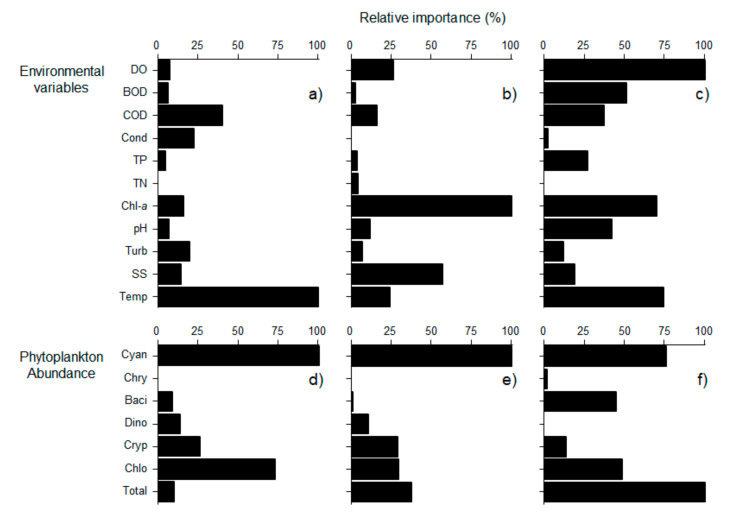
Relative importance (%) of environmental variables (**a**–**c**) and phytoplankton abundance (**d**–**f**) in predicting category with highest geosmin concentration (D) in three different depths. Surface layer (**a**,**d**), Middle layer (**b**,**e**), and, Bottom layer (**c**,**f**). Abbreviation of each variable indicated in Table 2.

The sensitivity analysis was conducted to evaluate the contribution of environmental variables and phytoplankton abundance in predicting the geosmin concentration categories using the MDL values in the RF model (Figure 6 and Figure 7). The relative importance of environmental variables and phytoplankton abundance was different for each layer. The abundance of Cyanophyceae exhibited relatively higher importance in its contribution to the prediction of the four categories in all three layers, particularly so in the surface layer (Figure 6). Among environmental variables, temperature was the most important variable for the prediction of the four categories in the surface layer, whereas chlorophyll and pH were the most influential variables for the prediction of the four categories in the middle and bottom layers, respectively. Meanwhile, abundance of Bacillariophyceae was the most influential variable for the prediction of the four categories in the middle layer, and total abundance showed relatively higher importance in the bottom layer.

In addition, when only the category with highest geosmin concentration was predicted, a similar pattern as that for the prediction of the four categories was observed. Cyanophyceae was the most influential variable in the surface and middle layers (Figure 7). In the bottom layer, total phytoplankton abundance showed relatively high importance. Among environmental variables, temperature was relatively important in the surface and bottom layers, whereas DO was the most influential variable in the bottom layer (Figure 7).

## 4. Discussion

In the process of proliferation, some algae produce various types of toxin materials and metabolites that cause earthy/moldy taste and odor such as geosmin and MIB [4,8]. Although they are not toxic at concentrations that can occur in water and fish and neither has been associated with serious health effects, they can cause tap water to smell and taste unpleasant [7]. In fact, Ömür-Özbek and Dietrich [38] reported that taste-and-odor problems became an issue after surface waters were used as drinking water sources in many places such as Europe, the Americas, and Japan [39,40]. Taste-and-odor metabolites cannot be removed by conventional water treatment operations such as coagulation and sedimentation. Thus, advanced water treatment processes like granular activated carbon (GAC) or ozone, which incur high financial costs, are used to remove these compounds [5,6,8]. Therefore, taste-and-odor compounds have been the subject of major interest in water management of water supply sources [3]. In this study, we analyzed the change in geosmin concentration at three different depths in the North-Han River watershed and its relationship with environmental variables and phytoplankton abundances using three different SDMs.

Geosmin concentration in the North-Han River watershed from May 2014 to December 2015 was categorized into four groups based on the boxplot method (Table 3), and the concentration of geosmin in group D, which shows the highest concentration, was found to be in the range of 10 ng/L or more. Taste-and-odor compounds such as geosmin can be detected by human noses at 5–10 ng/L, although this varies from individual to individual [41]. Accordingly, the drinking water quality standard in Japan has set the acceptable concentration of geosmin at 10 ng/L or less [42], whereas Korea manages its geosmin concentration below 20 ng/L [14].

Of the SDMs, each modeling approach used in the analysis of ecological data has its strengths and weaknesses, and seemingly only small differences exist in the accuracies of the models [28,43]. In this study, multiple criteria, such as accuracy and AUC, were applied to solve these problems and to select the most suitable model for predicting geosmin concentration categories. Indeed, the predictive performances of the three SDMs were different (Table 4). Among the three SDMs in this study, the RF model showed the highest prediction power for the geosmin categories in terms of both environmental variables and phytoplankton abundance. In particular, the AUC value of the RF model at all layers showed above 0.9 (Table 5), indicating that it was an excellent model according to Swets [31]. Similarly, Harris and Graham [2] reported that the RF model was the best-suited model for the prediction of geosmin concentration under 20 ng/L. As a prediction model, RF is a powerful tool for the analysis of ecological data due several advantages it possesses, such as high classification accuracy, a novel method of determining variable importance, and the ability to model complex interactions among predictor variables [44,45].

Cyanobacteria blooms mainly cause abnormal growth of phytoplankton, but can seriously affect drinking water supply due to cyanobacteria metabolites such as toxins and taste substances [2]. In our results, geosmin concentrations were shown to be high during the summer, when phytoplankton abundance increased due to cyanobacteria blooms (Figure 4 and Figure 5). Generally, *Anabaena* spp., major blue-green algae, are the most representative species producing geosmin worldwide [46]. In particular, cyanobacteria such as *Anabaena*, *Aphanizomenon*, *Oscillatoria*, and *Microcystis* are the main group of microorganisms that are responsible for the earthy-musty odor in drinking water, and are mostly found as surface scum and benthic mat in eutrophic waters [47,48,49,50]. In particular, according to previous studies [51,52], *A. circinalis* had proliferated and had been observed in the form of scum in the surface layer of Paldang Lake after July 2014; consequently, the concentration of geosmin had rapidly increased. After August 2014, however, the geosmin concentration had sharply decreased due to the dilution effect caused by the confluence of upstream and seasonal rain, combined with the flushing effect caused by the opening of the dam [53,54].

Meanwhile, temporal changes in both the phytoplankton community and geosmin concentration according to depth were different for each lake (Figure 3 and Figure 4). The change in geosmin concentration has been shown to be positively related to the amount of *A. spiroides* [55]. This was reflected in our study, in that the relative importance of Cyanophyceae abundance was highest in the RF model for predicting the categories of geosmin concentration (Figure 6 and Figure 7). The relative importance of temperature was also the highest among the environmental factors in the surface layer of the lake in this study (Figure 6 and Figure 7), because the decrease of water temperature had the greatest effect on the reduction of *A. spiroides*. The dominant period of Cyanophyceae can be determined by the trophic state and water temperature of the lakes, and low water temperature in winter is a major contributor to the extinction of Cyanophyceae [56,57,58]. Furthermore, turbidity, which was relatively high in terms of importance in the bottom layer, exhibited such high importance due to its relationship with the light environment for algae growth [4]. These depth-variable environmental variables are likely to be the key factors driving changes in the composition of the phytoplankton community [59].

## 5. Conclusions

In this study, the RF method demonstrated the best predictive power for geosmin concentration categories in the surface layer of lakes among the three machine-learning techniques (MARS, CART, and RF). Therefore, in the RF model approach for the further analysis (i.e., estimating the geosmin concentration categories with environmental variables and phytoplankton abundance at different lake depths), the model showed higher prediction in case of only predicting the highest geosmin concentration category compared with predicting four categories based on the boxplot. The sensitivity analysis of the model showed that temperature and Cyanophyceae abundance were highly important in the prediction of geosmin concentration categories in the surface layer, whereas total phytoplankton abundance was important for predicting geosmin concentration categories in the bottom layer.

## Figures and Tables

**Table 1:** Information of morphometric and hydrological characteristics in three lakes in the study.

Factors	Lakes		
	Uiam	Cheongpyeong	Paldang
Watershed area (km^2^)	7709	9921	23,800
Total storage (10^6^ m^3^)	80	185.5	244
Effective storage (10^6^ m^3^)	57.52	82.6	18
Inflow (10^6^ m^3^/year)	5323	6837	17,020
Outflow (10^6^ m^3^/year)	5322	6836	16,988
Dam height (m)	23	31	29
Residence time (day)	7.3	9.3	12.9

**Table 2:** Summary of 19 variables used in the prediction model (units, methods of measurement and abbreviations).

Variables		Abbreviation	Units	Methods
Independent variables	Cyanophyceae	Cyan	cell/mL	-
	Chrysophyceae	Chry	cell/mL	-
	Bacillariophyceae	Baci	cell/mL	-
	Dinophyceae	Dino	cell/mL	-
	Cryptophyceae	Cryp	cell/mL	-
	Clorophyceae	Chlo	cell/mL	-
	Total abundance	Total	cell/mL	-
	Water Temperature	Temp	°C	Multimeter in the field
	Conductivity	Cond	μS/cm	
	Turbidity	Turb	NTU	
	pH	pH	-	
	Dissolved Oxygen	DO	mgO_2_/L	
	Chemical Oxygen Demand	COD	mgO_2_/L	COD_Mn_
	Biochemical Oxygen Demand	BOD	mgO_2_/L	Membrane Electrode Method
	Total Phosphorous	TP	mg/L	Ascorbic acid analysis
	Total Nitrogen	TN	mg/L	Ascorptiometric analysis
	Suspended Solid	SS	mg/L	GF/C
	Chlorophyll *a*	Chl-*a*	μg/L	Ascorptiometric analysis
Dependent variables	Geosmin	-	ng/L	HS-SPME

**Table 3:** Categories of geosmin concentration were defined based on the boxplot method. The numbers in parenthesis (*n*) indicate the number of sampling sizes.

Category	A (≤25%)	B (25–50%)	C (50–75%)	D (>75%)
Range of geosmin (*n*)	≤4 (45)	4–6 (28)	6–10 (34)	>10 (34)

**Table 4:** Comparison of the predictive performance of three different models with different combinations of independent variables (environmental variables and phytoplankton abundance). CART: classification and regression tree, MARS: multivariate adaptive regression splines, and RF: random forest. Categories (A–D) of geosmin concentration are given in Table 3.

Dataset	Model	Environmental Variables		Phytoplankton Abundance	
		AUC	Accuracy	AUC	Accuracy
Four categories (A–D)	MARS	0.724	0.553	0.623	0.447
	CART	0.761	0.617	0.713	0.574
	RF	0.974	0.809	0.934	0.681
Category with highest geosmin concentration (D)	MARS	0.904	0.809	0.780	0.745
	CART	0.790	0.830	0.816	0.787
	RF	0.969	0.872	0.914	0.851

**Table 5:** Prediction accuracy of geosmin concentration in three different water layers using random forest model. Categories (A–D) of geosmin concentration are given in Table 3.

Dataset	Layer	Environmental Variables		Phytoplankton Abundance	
		AUC	Accuracy	AUC	Accuracy
Four categories (A–D)	Surface	0.974	0.809	0.934	0.681
	Middle	0.976	0.766	0.923	0.745
	Bottom	0.981	0.787	0.937	0.617
Category with highest geosmin concentration (D)	Surface	0.969	0.872	0.914	0.851
	Middle	0.967	0.851	0.906	0.787
	Bottom	0.981	0.830	0.920	0.830
